# Optimized Co-extraction and Quantification of DNA From Enteric Pathogens in Surface Water Samples Near Produce Fields in California

**DOI:** 10.3389/fmicb.2018.00448

**Published:** 2018-03-13

**Authors:** Michael B. Cooley, Diana Carychao, Lisa Gorski

**Affiliations:** Produce Safety and Microbiology Research Unit, Agricultural Research Service, United States Department of Agriculture, Albany, CA, United States

**Keywords:** droplet digital PCR, surface water, pathogen, gram positive, gram negative, quantification

## Abstract

Pathogen contamination of surface water is a health hazard in agricultural environments primarily due to the potential for contamination of crops. Furthermore, pathogen levels in surface water are often unreported or under reported due to difficulty with culture of the bacteria. The pathogens are often present, but require resuscitation, making quantification difficult. Frequently, this leads to the use of quantitative PCR targeted to genes unique to the pathogens. However, multiple pathogen types are commonly in the same water sample, both gram + and gram –, leading to problems with DNA extraction. With Shiga toxin-producing *Escherichia coli* (STEC), *Salmonella enterica* and *Listeria monocytogenes* as target, a method was optimized to co-extract all three and quantify the level of each using droplet digital PCR (ddPCR). Multiplexed target genes in STEC were virulence genes, shiga toxin 2 (*stx*2) and hemolysin (*ehx*). Likewise, multiplexed targets in Listeria and Salmonella were the virulence genes listeriolysin (*hly*) and invasion protein A (*inv*A). Water samples were processed using microbiological techniques for each of the pathogens and duplicate water samples were quantified by ddPCR. A significant correlation was found between culture and ddPCR results indicating detection primarily of culturable cells by ddPCR. Average virulence gene levels were 923, 23 k, 69 and 152 copies per sample for *stx*2, *ehx, hly* and *inv*A, respectively. Additionally, *stx*2, *ehx* and *inv* levels were significantly correlated (*P* < 0.05, *R* = 0.34) with generic *E. coli* MPN levels in the duplicate samples. Indirect quantification with ddPCR will improve understanding of prevalence of the pathogens and may reduce risks associated with contaminated surface water.

## Introduction

Vegetables are a common source of foodborne illness in the United States and elsewhere, primarily because several produce varieties are consumed raw. In fact, nearly half of the outbreak-associated foodborne illnesses in the United States are leafy vegetables and, additionally, many sporadic illnesses are linked to produce (Crowe et al., 2015; Henao et al., 2015). Produce can become contaminated at any point in the production chain, yet pre-harvest contamination is prevalent and difficult to prevent, as evidenced by the 2006 spinach outbreak and subsequent outbreaks (Anonymous, 2006; Allerberger, 2009). Surface water, such as rivers, lakes and ponds can provide a reservoir for the pathogens (Hanning et al., 2009; Lynch et al., 2009; Oliveira et al., 2011). The water can become contaminated from a variety of sources such as exposure to wildlife, sewage, and agricultural runoff from animal operations (Gagliardi and Karns, 2000; Walters et al., 2011). In turn, wildlife can become contaminated through exposure to contaminated water, with subsequent deposit of pathogens via feces onto fields (Fenlon, 1985; Kirk et al., 2002; Jay et al., 2007; Gorski et al., 2013).

The majority of bacterial foodborne illnesses and recalls associated with produce are due to Shiga toxin-producing *Escherichia coli* (STEC) and *Salmonella enterica* (Crowe et al., 2015). Additionally, *L. monocytogenes* contamination of produce recently has led to several high profile outbreaks (Doell, 2010; Anonymous, 2011, 2013). During a survey of several public watersheds in the central coastal California to determine the prevalence of STEC, *Salmonella*, and *L. monocytogenes*, we recognized the need for pathogen quantification. High incidence at select locations suggests high levels of contamination, yet the actual contamination levels in the watersheds are not reported (Cooley et al., 2014). Since enteric bacteria in the watersheds experience various levels of stress, most will not produce colonies without resuscitation leading to an under-estimation of pathogen levels (Buerger et al., 2012). Typical resuscitation (enrichment) will produce an unknown number of cell divisions depending on the cell physiology, making direct plating unsuitable for quantification. Quantification methods are available which utilize enrichment and Most Probable Number (MPN) determination by either culture methods or PCR methods (Mcegan et al., 2013; Orlofsky et al., 2015; Benami et al., 2016). However these methods are labor intensive, especially if a large number of samples is involved. Quantitative PCR (QPCR) targeted to virulence genes is a more rapid method (Parsons et al., 2016; Shridhar et al., 2016; Yergeau et al., 2016; Weber et al., 2017). Furthermore, a new type of QPCR, called droplet digital PCR (ddPCR) is more efficient and less sensitive to PCR inhibitors (Racki et al., 2014; Verhaegen et al., 2016).

The survey mentioned above discovered hundreds of samples positive for STEC, *L. monocytogenes* or *S. enterica*, as previously reported. The microbiological methods used during this survey are very sensitive to the presence of the pathogens, i.e., dual and parallel isolation methods have improved prevalence (Pritchard and Donnelly, 1999; Gorski et al., 2011) and, in the case of STEC, sensitivity is less than 10 cells per sample (Cooley et al., 2013, 2014). If ddPCR is sufficiently sensitive, pathogen presence from ddPCR should correlate to prevalence data already reported from these samples. Nevertheless, QPCR data representative of each pathogen level in a sample requires efficient extraction and amplification of DNA from each of the pathogens. However, the DNA of *L. monocytogenes*, like most gram positive organisms, is difficult to extract with the same efficiency as gram negative organisms (Krakat et al., 2017). This problem impacts not only QPCR of pathogens but also several other quantification methods, such as metagenomics, where samples are known to include a host of unknown organisms and probably both gram + and gram –bacteria. Consequently, we include a study of several extraction methods using spiked samples in an attempt to achieve a balanced extraction from these complex samples.

## Materials and methods

### Summary of swab sampling techniques and locations

**Figure 1:**
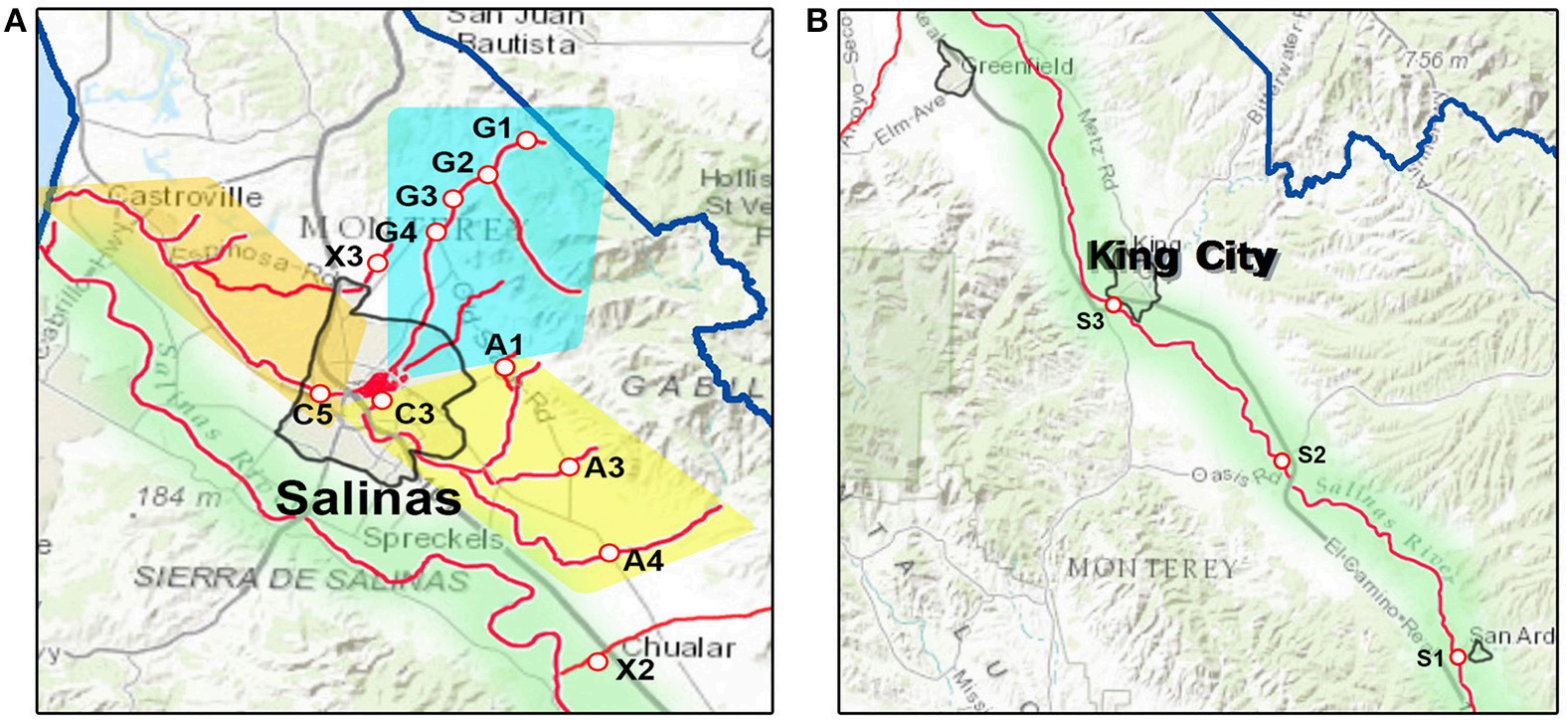
Maps of sampling sites and watersheds in Monterey County, including; **(A)**, sampling sites near the city of Salinas and **(B)**, sampling sites near King City. The waterways are marked as redlines. The sampling sites are labeled with a letter corresponding to the watershed to which they have been assigned and a number to differentiate between sites within that watershed. A, Alisal Creek; C, Carr Lake; G, Gabilan Creek; S, Salinas River, X, extraneous (no designated watershed).

Sampling sites in Monterey County in California were selected on the basis of ease of access and have been sampled repeatedly in the last 12 years using Moore swabs (see below), deployed for 24 h at the sites in Figure 1 (Cooley et al., 2007, 2014). Sites were grouped into regions based on watershed when possible. Carr Lake is likely impacted by seepage from septic systems of Salinas. Conversely, upstream regions (Gabilan Creek, Alisal Creek and the upper portion of the Salinas River are animal-impacted as they were exposed, to a great extent, by wildlife in riparian areas, and runoff from cattle ranches (primarily cow-calf operations).

### Microbiology methods

At 2-week intervals over 10-months (12/15–9/16), duplicate Moore swabs (cut cheesecloth, gathered and tethered on a single fishing line) were deployed for 24 h at the above sample sites. Swabs were placed into Whirl-Pak bags (Nasco), kept on ice during transport and one of the duplicate swabs for each location was frozen at −80°C. For the remaining swab, 500 mL of sterile water was added to each bag, followed by vigorous shaking by hand for 20 s to recover a representative sample of the sediment. From the swab eluate, 100 mL was removed for *L. monocytogenes* isolation, and 100 mL was removed for generic *E. coli* quantification. Most probable number (MPN) quantification of generic *E. coli* was determined by the Colilert QuantiTray 2000 method according to the manufacturer's recommendations (Idexx Laboratories). To the swab and remaining eluate in the Whirl-Pak bag, 30 mL of 10X Tryptic Soy Broth (TSB) was added, and the bag was incubated with shaking at 200 RPM at 25°C for 2 h, then 42°C for 8 h. The TSB-enriched cultures were used for STEC and Salmonella isolations. O157 STEC and non-O157 STEC were isolated by methods published previously (Cooley et al., 2013). Briefly, genomic DNA was heat-released from TSB enrichment and QPCR was performed using a multiplexed primer set to detect all shiga toxin (*stx*) types. The QPCR-positive TSB-enriched cultures were streaked onto CHROMagar O157 media plates (DRG International) and isolated, mauve, *E. coli*-like colonies were selected for a second round of QPCR using the same *stx* multiplex primer set. In a parallel procedure, TSB-enriched culture was subjected to Immuno Magnetic Separation (IMS) with anti-O157 antibody (Invitrogen/Dynal), and the IMS beads were spread on two types of media; modified sheep blood agar (mSBA), novabiocin and tellurite Rainbow agar (NT-RA) (Biolog) (Cooley et al., 2013). All plates were incubated at 37°C for 24 h. Suspected O157:H7 colonies were selected on the basis of colony color, and were analyzed by PCR for the presence of the O157 O-antigen synthesis (*rfb*E) and intimin (*eae*) genes (Cooley et al., 2007). Similarly, non-O157 *E. coli*-like colonies were selected from NT-RA (red colonies) and mSBA (blue colonies that showed hemolytic activity) and confirmed by real-time PCR using the *stx* multiplex primer set described above.

The same TSB enriched culture was also used for Salmonella isolation in two parallel procedures (Kalchayanand et al., 2009). Portions of the TSB-enriched culture was either subject to IMS with anti-Salmonella antibody (Dynal, Invitrogen) followed by Rappaport- Vasilliadis Soya Peptone Broth (RVS, Oxoid, Remel) or plated onto Modified Semi-solid Rappaport Vasilliadis (MSRV) medium. Colonies from both RVS and MSRV were streaked onto Xylose Desoxycholate agar (XLD, Difco, Becton Dickinson-BBL). Isolated black colonies on XLD were picked and were confirmed as Salmonella by PCR for the *inv*A gene (Gorski et al., 2011, 2013).

Enrichment and isolation of *L. monocytogenes* was performed as described previously (Cooley et al., 2014). Swab aliquots were enriched with Buffered Listeria Enrichment Broth Base (BLEB, Difco) for 18 h at 30°C and subsequently subjected to IMS with anti-Listeria antibody (Dynal, Invitrogen), with two parallel methods used to detect *L. monocytogenes* from the beads. Aliquots of re-suspended beads were inoculated into Fraser Broth and incubated at 37°C. Isolated blackened media colonies were sub-cultured onto Brilliance Listeria Agar plate (Oxoid, Remel). A separate aliquot of the re-suspended beads was plated onto Brilliance Listeria Agar and incubated for 2 days at 37°C. Isolated blue colonies surrounded by clearing were picked and streaked onto Modified Oxford Agar (MOX). Bluish-white colonies from MOX and Brilliance were selected for detection of the *hly*A gene by PCR (Cooley et al., 2014).

### TAQman primer design method

Examination of the published sequences of *stx*2 and *ehx* variants in STECs in GenBank revealed conserved regions for designing PCR primers and probes. Likewise, Salmonella and Listeria sequences for *inv*A and *hly* were examined for conserved regions, respectively. Probes for Salmonella and Listeria were designed with an internal Nova quencher to allow for smaller probe sequence and lower base fluorescence (BioSearch). Restriction patterns were also considered to eliminate those regions where the restriction site HindIII was located within the amplicons. Likewise a unique region of the plasmid pHCred (Takara Corp) was selected as internal control (IC) within the coding region of the fluorescent protein from sea anemone *Heteractis crispa*. All primers and probes were examined to minimize internal hairpin and dimer formations with itself and other members of the multiplex. Primer length and/or position were also adjusted to allow optimal amplification with 60°C annealing temperature for all multiplex sets. Sequence of the selected primers and probes are listed in Table 1. Multiplex sets were constructed as STEC (*stx*2 and *ehx*), Sal/Lm (*inv*A and *hly*), IC (*stx*2, IC). Primer and probe sequences were BLASTN at NCBI to ensure they are unique to their respective targets.

**Table 1:** Primers and probes.

**Multiplex**	**Name**	**Sequence**
STEC	Stx2 forward	GGACCACATCGGTGTCTGTTATT
	Stx2 reverse	CCCTCGTATATCCACAGCAAAAT
	Stx2 probe	HEX-CCACACCCCACCGGCAGT-BHQ1^a^
	Ehx forward	TTATCGACAACAGCTGCAAGTG
	Ehx reverse	GCTTAGCTCGCTCAAATTTATCTG
	Ehx probe	FAM-CGGCTGTTATGCTGGCTATCAGTCCTCTT-BHQ1
Sal/Lm	InvA forward	GGCGGTGGGTTTTGTTGTCTTCTCTATTGTCA
	InvA reverse	CTGTTTACCGGGCATACCATCCAGAGAAAATC
	InvA probe	FAM-CGACTTCCG-Nova^b^-CGACRCGTTCTGAACCTTTGGTAA-BHQ1
	Hly forward	ACCAGCATCTCCGCCTGCAAGTCCTAAG
	Hly reverse	CTTTTCTTGGCGGCACATTTGTCACTGC
	Hly probe	HEX-CCAATCGAA-Nova-AAGAAACACGCGGATGAAATCGATA-BHQ1
Internal Control	IC forward	AACGTGATGGCCCTGAAGGT
	IC reverse	CTGGCCTCGTACAGCTCGAA
	IC probe	FAM-ACTCGTCCTTCTTCTTCCGCAGCATCT-BHQ1

a *Black Hole Quencher 1 (BioSearch)*.

b *Nova, internal quencher (BioSearch)*.

### DNA extraction procedure

Duplicate frozen swabs, mentioned above, were thawed in 190 mL sterile water. Representative samples of the sediments in the swabs were recovered by agitation (1 min vigorous agitation by hand). Liquid was moved into sterile conical tubes and centrifuged (Sorvall) swinging bucket rotor (4,700 RPM) for 10 min at room temp. The pellet was weighed and 2–3 gm was extracted using the DNeasy PowerMax Soil Kit (MoBio Corp) following the manufacture's protocol. For extractions from samples potentially containing Listeria, different pre-treatments prior to the original PowerSoil Kit protocol were used, including lysozyme (Sigma, 10–70 mg/mL), mutanolysin (Sigma, 250 U/mL), lysostaphin (Sigma, 40 U/mL) with extended incubations at 37°C (1–3 h), beadbeating (garnet or glass beads, 1 min full power (BioSpec Mini-8), sonication (2 min at power level 4, 10 s pulses, Virtis Virsonic 550 with CV4 20 KHz converter and microprobe (3 mm). Sonication was performed in a SterilGARD III biosafety cabinet (Baker Co.). Extraction optimization and sensitivity testing was done by spiking *E. coli* O157 (RM1484), *S. enterica* (RM7323) and *L. monocytogenes* (RM2194) into swab pellets. Number of spiked cells was determined by plate count. Purified DNA was quantified using Nanodrop (ThermoFisher).

### ddPCR procedure

Droplets for Droplet Digital PCR (ddPCR, BioRad) were created following the manufacture's protocol for 20 μL reactions using 10 μL BioRad's Supermix for Probes, 2 μL primer (0.3 μM) and probe (0.2 μM) (10X mix), up to 1 μg DNA, 1 μL internal control (1 fg/μL, pHCred, Takara), 1.2 μL MgCl_2_ (25 mM stock), 0.2 μL HindIII (20 U/μL). Droplets were created with Droplet Generation Oil for Probes in the QX200 droplet generator (BioRad), then transferred to a 96 well PCR plate and amplified in a thermal cycler 5 min at 95°C, 45 cycles at 95°C for 30 s and 60°C for 90 s, then 5 min at 72°C, 5 min at 98°C, 4°C overnight. Pathogen genomic DNA with their respective target gene(s) was spiked into reactions containing 100–1000 ng sediment DNA. A single copy of the target gene is expected within these genomes. Sediment samples used for these spiking experiments were previously shown to be negative for pathogen(s) using the culture method described above. Droplets were processed with the QX-200 Droplet reader and the QuantaSoft software version 1.7.4 (BioRad) using the Rare Event Detection procedure.

### Statistical analysis

Confidence interval for predicted template levels was computed by QuantaSoft (BioRad) using Poisson distribution statistics. In most cases the threshold fluorescence level for positive droplet detection was automatically determined by a proprietary algorithm within QuantaSoft. Manual threshold was determined as necessary using automatic settings within duplicate or control wells as per BioRad Best Practices Guidelines. Correlations between ddPCR results (pathogen presence or absence) and culture results used the Dice Coefficient in AddinSoft XLSTAT Base version 2018. One Way ANOVA was used to determine the significance between log_10_ transformed ddPCR results and both sample watersheds and sample seasons. Pearson Product Moment was used for correlation analysis between log_10_ transformed ddPCR results and log_10_ transformed 5 day prior precipitation totals and log_10_ transformed generic *E. coli* MPN values. Both One Way ANOVA and Pearson Product Moment were from SigmaPlot version 11.0

## Results

### Internal control development

**Figure 2:**
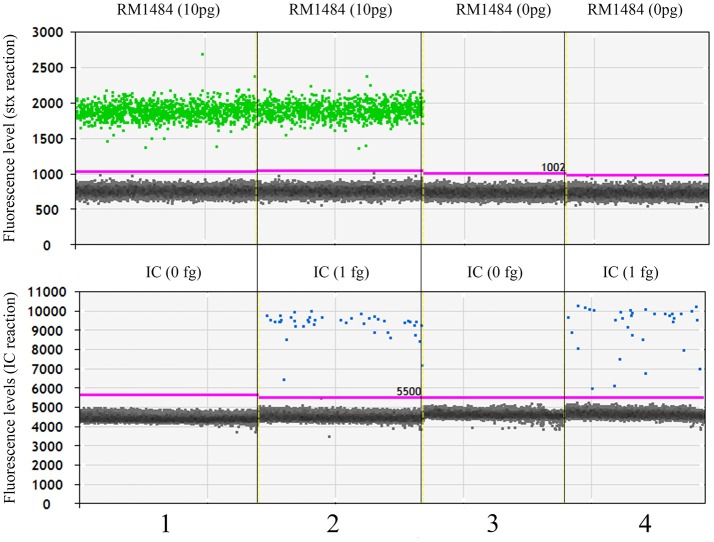
Representative ddPCR display of multiplexed *stx*2, IC amplifications. Most droplets displayed basal fluorescence of 750 and 4,500 units for the *stx*2 and IC reactions, respectively. Threshold levels are marked in pink and those droplets above these levels are considered positive for the respective amplicon; green, *stx*2 positive, blue, IC positive.

Since sensitivity of pathogen detection is dependent on the amount of swab DNA added to the reactions, initial studies looked at the effect of non-target DNA levels on the ability to detect IC and the O157 strain RM1484 with *stx*2 as target. Non-target DNA added to these reactions came from swabs, the duplicate of which had previously been shown to contain no detectable pathogen using microbiological methods. The culture methods (<10 cells/swab for STEC) are very sensitive. Nevertheless, these swabs may contain very low levels of the targeted pathogen. Spiked reactions included 1 fg IC molecules or 10 pg of RM1484 for the *stx*2 target (Figure 2). At both 100 ng and 1 μg non-target swab DNA per reaction, ddPCR could detect all the spiked *stx*2 genes (Table 2). In contrast, only a fraction (29%) of spiked IC was detectable. Neither IC nor *stx*2 was detected without the corresponding DNA spike, indicating the absence of *stx*2 in this swab DNA and the absence of IC in swab and RM1484 DNAs. One microgram of DNA is the upper limit on the amount of DNA per 20 μl ddPCR reaction recommended by BioRad. All future ddPCR reactions will be at the 1 μg DNA level and produced in triplicate.

**Table 2:** The effect of DNA levels on IC and *stx*2 detection.

**Reaction**	**IC spike^a^**	**O157 spike^b^**	**Total DNA^c^**	**IC exp^d^**	***stx*2 exp^e^**
1	0	0	100 ng	0	0
2	0	2126	100 ng	0	2720
3	276	0	100 ng	72	0
4	276	2126	100 ng	84	2795
5	0	0	1 μg	0	0
6	0	2126	1 μg	0	2740
7	276	0	1 μg	78	0
8	276	2126	1 μg	87	2705

a *IC spike level (molecules per reaction) based on known MW of the pHCred plasmid*.

b *E. coli O157 (RM1484) genomic DNA, number of molecules/reaction, based on 5.1 × 10^6^ bp*.

c *DNA per reaction, primarily sediment DNA from a pathogen-negative sample*.

d *IC template levels per reaction as detected by ddPCR, average of four experiments*.

e *stx2 template levels per reaction as detected by ddPCR, average of four experiments*.

### DNA extraction optimization

As a further test of the sensitivity of the ddPCR protocol, all three pathogens were spiked into sediment pellets recovered from swab samples previously shown to be negative for these pathogens. Cells were spiked at 10^4^ CFU per pellet and DNA was extracted following the basic MoBio protocol. Sensitivity demonstrated by the ddPCR reactions ranged from 0.4 (*ehx*) to 0.015 (*hly*) (Table 3). Amplification of IC was the same as above and did not show PCR inhibition as factor in these reactions (data not shown). Nevertheless, the relative insensitivity of the PCR reactions and/or poor recovery of DNA from the spiked cells was indicated. Poor DNA recovery was especially indicated for Listeria (*hly*) since preliminary experiments with spiked *L. monocytogenes* DNA did not indicate a *hly* reduction (data not shown). Advice from MoBio and literature review indicated several DNA extraction remedies. However, all of these remedies were adapted from extraction protocols on pure culture; very different from extraction of DNA from 10^4^ cells from sediment. Nevertheless, each of these implemented protocol changes was an improvement over the basic MoBio method (Table 3). However, only including sonication in the method brought *hly* detection near to the level of *L. monocytogenes* cells inoculated into the pellet.

**Table 3:** Extraction method improvements.

**Method**	**Spiked cell detection^a^ (%)**			
	**stx**	**ehx**	**invA**	**hly**
MoBio Basic	15	40	13	1.5
+ Lysozyme	49	86	26	1.1
+ Enzyme mix^b^	27	42	24	6
+ Beadbeat (BB)	83	133	28	5
+ Enzyme mix + BB	118	160	45	8
+ Sonication	92	83	58	101

a *STEC, Salmonella and Listeria were spiked into culture-minus swab samples at 10^4^ CFU/swab and subsequently DNA was extracted by the methods listed. The spike was detected by ddPCR using the virulence gene indicated. Each number is the average of 3 reactions*.

b *Enzyme mix included lysozyme, mutanolysin and lysostaphin*.

### Sensitivity of the ddPCR procedure

The sonication/extraction method described above was subsequently used with a series of swab pellets spiked with different pathogen levels. Target genes were quantified in these DNAs to indicate the sensitivity of the ddPCR reactions (Table 4). The ddPCR method was sensitive to spiked cells to the lowest level tested, 10 cells per swab of each pathogen. The lowest fraction detectable was 5 Listeria cells (*hly*). All other targeted genes were detected at the spiked level (10 cells). Higher spiking levels were also detected at or close to the spiked level, with the exception of the Salmonella 1,000- and 100-cell spikes, detected at 380 and 45 *inv* genes, respectively. Without the spiked pathogens, the targeted genes were not detected in the swab pellets, indicating that DNA from the indigenous microflora in the pellets was not interfering with the method, even at low pathogen levels.

**Table 4:** Sensitivity test using final sonication method.

**Target gene**	**Fraction of spiked cells quantified by ddPCR^a^ (SE)^b^**			
	**1,000 cells**	**100 cells**	**10 cells**	**No cells**
*stx*2	0.92(0.13)	0.98(0.04)	1.05(0.25)	0(0)
*ehx*	1.05(0.08)	1.15(0.23)	1.51(0.19)	0(0)
*inv*	0.38(0.01)	0.45(0.05)	1.01(0.42)	0(0)
*hly*	0.99(0.07)	0.95(0.12)	0.48(0.05)	0(0)

a *Cells of RM1484, RM7323 and RM2194 were spiked together into swab pellets at the indicated cell levels, DNA extracted and quantified by ddPCR for the indicated genes. Quantifications are the average of 6 independent extractions and ddPCR reactions*.

b *SE, Standard Error*.

### Comparison of ddPCR and incidence in swabs

Using the above optimized DNA extraction technique, 36 swabs were processed and the DNA used for ddPCR with the two multiplex primer sets to detect virulence genes in STEC, Listeria and Salmonella. Presence or absence of the target virulence genes from the 36 reactions were compared with microbiological results from the duplicate swabs (Table 5). Best correlation was found with *inv* amplification (Dice 87.7%). Nevertheless, all virulence genes significantly correlated with their respective culture results. The range of quantification for individual genes varied considerably between samples, with the greatest template variation (0–753 k) and least correlation (Dice 75%) occurring with the gene *ehx*. Remarkably, there were no differences between pathogen levels and sample watersheds (Figure 1) or sample season (winter, spring, summer, or fall) (Table 6). Also, pathogen levels were not correlated with rain levels (5 day prior). However, *stx*2, *ehx*, and *inv* levels were significantly correlated with generic *E. coli* (Table 5).

**Table 5:** Quantification of environmental pathogens in Moore swabs by ddPCR.

**Organism**	**Target gene**	**Culture positive**	**PCR positive**	**Ave template level (range)**	**Dice similarity coefficient^a^ (%)**	**Correlation to generic *E. coli***
Shiga toxin-producing *E. coli* (STEC)	*stx*2	19/36	21/36	923 (0–20 k)	85	*P* = 0.04, *R* = 0.34
	*ehx*	19/36	29/36	23 k (0–753 k)	75	*P* = 0.03, *R* = 0.38
Listeria	*hly*	22/36	21/36	69 (0–362)	79.1	*P* = 0.09, *R* = 0.29
Salmonella	*inv*	27/36	30/36	152 (0–517)	87.7	*P* = 0.03, *R* = 0.36

a *Comparing ddPCR positive and culture positive samples*.

**Table 6:** ddPCR quantification in individual Moore swabs.

**Sample**	**Sample location^a^**	**Sample date**	***Template levels per swab***				**5 days rain^b^**	**generic *E. coli MPN/swab***
			***stx*2**	***ehx***	***hly***	***inv*A**		
FN2569	A3	12/28/15	78	171	0	0	0.29	1,286
FN2611	A3	1/13/16	160	374	0	0	0.01	77,000
FN2628	S1	1/26/16	7	20	8	8	0.14	839
FN2634	G2	1/26/16	83	111	83	0	0.66	21,560
FN2656	S1	2/10/16	21	575	0	0	0.00	2,129
FN2657	S2	2/10/16	0	123	0	41	0.00	12,319
FN2683	S1	2/26/16	14	28	0	17	0.01	479
FN2713	S3	3/8/16	336	10,126	249	517	1.58	572,660
FN2717	A1	3/8/16	717	4,995	86	86	1.50	2,156,880
FN2746	G2	3/22/16	248	331	137	128	0.21	13,640
FN2753	C3	3/22/16	0	279	237	146	0.21	1,599,400
FN2755	C5	3/22/16	0	767	185	175	0.21	1,694,220
FN2768	G1	4/6/16	36	22	0	0	0.00	7,554
FN2796	S2	4/20/16	0	108	101	17	0.00	4,400
FN2798	A4	4/20/16	0	90	126	109	0.00	110,880
FN2800	G2	4/20/16	104	104	39	78	0.00	26,400
FN2852	G3	5/18/16	387	571	0	306	0.00	91,080
FN2853	G4	5/18/16	145	456	362	870	0.00	697,180
FN2872	S1	6/1/16	58	36	27	165	0.00	60,500
FN2877	G2	6/1/16	0	0	53	166	0.21	42,460
FN2879	G3	6/1/16	0	185	149	298	0.21	144,540
FN2898	S1	6/15/16	73	194	0	296	0.00	16,280
FN2906	G4	6/15/16	0	52	45	89	0.12	100,100
FN2945	S1	7/6/16	38	0	0	239	0.00	4,260
FN2947	X2	7/6/16	0	0	155	265	0.00	8,459
FN2949	A3	7/6/16	0	3372	145	289	0.00	447,700
FN2950	G2	7/6/16	0	127	88	278	0.00	9,020
FN3022	A4	8/16/16	0	0	61	189	0.00	44,220
FN3026	G3	8/16/16	0	0	0	18	0.02	35,200
FN3046	A3	8/30/16	6382	13,851	0	0	0.00	2,647,260
FN3049	G3	8/30/16	210	256	0	28	0.00	110,880
FN3050	X3	8/30/16	0	0	0	27	0.00	29,040
FN3068	A3	9/13/16	411	28,381	0	373	0.00	572,660
FN3069	G2	9/13/16	3428	8,041	119	63	0.00	257,180
FN3091	A3	9/27/16	20309	7,53,492	0	106	0.02	5,323,120
FN3095	X3	9/27/16	0	0	19	81	0.02	21,849

a *Refer to sample site designation in Figure 1*.

b *Cumulated precipitation 5 days prior to sampling (University of California, Integrated Pest Management Weather Database)*.

## Discussion

Pathogen level in moving water is very dynamic. This has been well established and does need quantification to demonstrate it. Previous and repeated sampling in the Salinas region has shown that even samples collected a few seconds apart at the same location can show the presence or absence of the pathogen (Cooley et al., 2007). With a sufficiently large number of samples, incidence data can help to define the level of contamination. Nevertheless, quantification does a better job of describing the nature of this environment, since each data point describes the level of contamination in that sample. Multiple samples are still needed, but there is a real benefit with quantification to those who develop models and risk assessments.

Quantification based on DNA comes with a few assumptions. One assumes complete extraction from the sample with sufficient purity for amplification. With environment samples this not always the case. The samples are often complex, containing unknown organisms. How is it that a single extraction procedure can portend to achieve a uniform and balanced extraction? In the process of establishing this ddPCR procedure it was found that Listeria DNA was poorly extracted, quite probably due to the stronger cell wall, compared to gram–bacteria (Krakat et al., 2017). Optimization was eventually achieved by sonication. Sonication shears the DNA but did not interfere with our TaqMan assay, probably due to the small size of the amplicons. This is evident by sensitivity to the target genes at (or close to) 10 cells per swab (Table 4). This research also investigated other methods including enzymatic digestion with 3 enzymes and bead beating. Other methods may still be possible with further research.

Since one purpose of this research is to develop ddPCR as a method to quantify the level of virulence genes in surface water and the high likelihood of PCR inhibitors in these samples (Tsai and Olson, 1992), it was necessary to first validate an internal PCR control (IC). The IC control selected was the gene HCred. HCred is a gene coding the red fluorescent protein from the sea anemone *Heteractis crispa* which is a gene not expected in these samples. With the samples in this research we have yet to find IC where is was not spiked into the reaction. Additionally, experience with ddPCR has shown a substantial insensitivity to PCR inhibitors (Singh et al., 2017), and also we have yet to find any sample with sufficient PCR inhibition to interfere with ddPCR.

Sensitivity of this assay is highly dependent on the amount of DNA added to the reaction. Unfortunately, the amount of DNA recovered from the sediment from one swab is usually so large that even 1 μg is but a small sampling. All the assays were in triplicate (3 μg total), nevertheless, if the target pathogen is very rare in the swab, it is easy to get a negative result from ddPCR and a positive from culture, despite the sensitivity of the ddPCR reaction.

Another assumption is that the DNA extracted from the sample is coming from the pathogen. This is especially an issue for *stx*2 since it has been shown to be present in phage. A percentage of the DNA may originate as phage or even naked DNA. Additionally, the copy number of the target genes may be greater than one per genome. Both of these issues would lead to over-estimation of number of pathogens present in the sample. Nevertheless, significant correlation was displayed between culture and ddPCR results (Table 5) indicating that a significant portion of virulence genes detected are coming from viable (and culturable) cells. It is noteworthy that the Dice coefficient, exhibited by *ehx* amplification and STEC culture results, was substantially smaller than with the other 3 virulence genes. It is very possible that non-STEC *E. coli* are present in these samples, many of which may contain the *ehx* gene. As such, *ehx* may be a comparatively weak indicator of STEC levels.

Reliance on incidence information in the past may have led to incorrect assumptions regarding the prevalence or spread of pathogens in the Salinas region. Previously, we had shown a strong seasonality of STEC incidence and presumably this is due to rainfall, since rainfall is greatest during the winter and early spring when STEC incidence is highest (Cooley et al., 2013). Likewise, both Listeria and Salmonella showed similar seasonality, though the effect with Salmonella was slight (Cooley et al., 2014). With the current research, target gene levels from winter/spring were statistically similar to those from summer/fall. Likewise, larger rainfall totals 5 day prior to the sample date failed to correlate to samples with elevated virulence gene levels. Also, sample sites which had previously been reported with high incidence for STEC (G1, G2, G3, and G4), Listeria (G2, G3) and Salmonella (G2, S3) were not statistically higher for the respective target genes compared other sample locations (Cooley et al., 2013, 2014). It would seem that assumptions made from incidence data will have to be re-visited with quantification data. Nevertheless, the number of samples processed by ddPCR in this research is substantially smaller in comparison to previous surveys. It may be necessary to process many more samples before reliable comparisons can be made.

## Author contributions

MC designed and executed the experiments, wrote the manuscript. DC executed the experiments; LG executed Listeria and Salmonella extraction experiments.

### Conflict of interest statement

The authors declare that the research was conducted in the absence of any commercial or financial relationships that could be construed as a potential conflict of interest.
